# Fatigue relief: the contribution of non-invasive neuromodulation (FAREviaNM)

**DOI:** 10.3389/fnins.2026.1735954

**Published:** 2026-04-10

**Authors:** F. Tecchio, L. Paulon

**Affiliations:** 1 Laboratory of Electrophysiology for Translational neuroScience (LET’S) – ISTC – CNR, Rome, Italy; 2 Independent Researcher, Engineer Freelance, Rome, Italy

**Keywords:** fatigue, neuromodulation, NIBS (non-invasive brain stimulation), personalized neuromodulation, transcranial direct Current Stimulation (tDCS)

## Abstract

Fatigue is a universal and constructive experience, yet chronic fatigue—defined as a persistent lack of energy that limits daily life as assessed by modified Fatigue Impact Scale (mFIS)—remains nowadays a symptom waiting for effective mitigation. Evidence across neurological, oncological, and psychosocial conditions shows that fatigue shares a common neurophysiological substrate: disrupted sensory-motor imbalances and network synchrony that can be targeted by established interventions—physical activity, mindfulness, yoga, and cognitive-behavioral therapy. Within this landscape, non-invasive neuromodulation stands out as an effective and safe tool to restore sensorimotor balance to integrate behavioral interventions. The Faremus protocol, based on five daily sessions of 15-min bilateral anodic transcranial Direct Current Stimulation (tDCS) over the somatosensory cortex, has shown consistent clinical benefits in multiple sclerosis: an average 26% reduction in fatigue severity, persisting for weeks or months, with excellent tolerability and home-use feasibility. Converging meta-analyses, neurophysiological central and behavioral investigations, and reviews confirm the reproducibility and mechanistic validity of this approach, positioning neuromodulation among the most promising evidence-based strategies in supporting fatigue relief. This perspective highlights neuromodulation as a transversal instrument to counter fatigue across conditions, and as a cornerstone of integrated, multidisciplinary strategies aimed at preserving brain plasticity, enhancing resilience, and restoring sustained well-being.

## That fatigue is mitigable redefines its scientific relevance

Fatigue is a universal human experience, typically arising as a physiological signal to rest after goal-directed activity. While physiological fatigue serves as a constructive feedback mechanism that protects from overexertion and directs to behaviors to restore energy balance, chronic fatigue is fundamentally different: it persists for months or years, substantially impairing daily functioning and quality of life. In this work, we define fatigue as a subjective yet measurable perception of persistent lack of energy, whose impact can be quantified using standardized scales, e.g., modified Fatigue Impact Scale (mFIS).” Far from being confined to one disease category, fatigue is recognized as a transversal condition, cutting across neurological, oncological, infectious, and psychosocial domains (Penner and Paul, 2017; Kuppuswamy, 2022; Raizen et al., 2023). It is consistently reported as one of the most distressing symptoms by people living with multiple sclerosis (MS), as a disabling comorbidity in cancer survivors, as a major impediment to rehabilitation after stroke or traumatic brain injury, and as a pervasive burden in the general population exposed to chronic stress or long-COVID (Kim, 2024). Global epidemiological analyses estimate that fatigue affects nearly 8% of the general population, with higher prevalence in women, in populations with lower socioeconomic status, and in Asian compared to American and European regions (Yoon et al., 2023). Emerging forms of cognitive and technological overload—often described as “AI-related fatigue” or technostress—further illustrate how sustained imbalance between cognitive demand and adaptive regulation may generate fatigue-like states beyond traditional medical conditions. The multiplicity of conditions in which it is generated and its global impact underscore the need to study fatigue as a phenomenon in its own right, rather than as a secondary outcome of the disease.

Efforts to conceptualize fatigue scientifically have long been hampered by its subjectivity. Nevertheless, reliable instruments have been developed and validated, allowing researchers and clinicians to capture its impact in quantitative terms. Among the most widely used are the Fatigue Severity Scale (FSS; Krupp et al., 1989) and the mFIS (Fisk et al., 1994; Mathiowetz, 2003). These scales establish standardized thresholds for clinically relevant fatigue and allow longitudinal monitoring of symptom progression (Strober et al., 2020). Despite inevitable limitations of self-reported questionnaires—including susceptibility to recall bias, cultural differences, and lack of standard physiological correlates—their broad adoption enables comparison across studies and patient populations. Importantly, advances in digital neurophysiology point toward the near-future availability of objective biomarkers, such as oculomotor signatures of saccadic dynamics, as candidate physiological measures of fatigue (Bafna and Hansen, 2021). Together, validated scales and emerging biomarkers make it possible to frame fatigue as a robustly defined and measurable condition, moving beyond vague or purely subjective descriptions.

A further critical step in establishing fatigue as a legitimate clinical and scientific target has been the demonstration that interventions exist to mitigate it. Four approaches consistently emerge from systematic reviews and meta-analyses as effective training tools across a wide range of conditions: Cognitive Behavioral Therapy—CBT (Deale et al., 1997; van den Akker et al., 2016), physical activity (Lee et al., 2012; Oberoi et al., 2018; Razazian et al., 2020), yoga (Ahmadi et al., 2013; Dong et al., 2019), and mindfulness-based interventions (Brown and Ryan, 2003; Ulrichsen et al., 2016). These approaches differ in theoretical background and implementation, yet they converge on the capacity to reduce perceived fatigue and improve quality of life.

The existence of reliable measures and validated interventions transforms the scientific landscape of fatigue. No longer dismissed as an inevitable consequence of illness or lifestyle, fatigue emerges as a legitimate target for neuroscience, physiology, and clinical innovation. Such a significant need—mitigating fatigue—guides therapeutic approaches, identifying available interventions at multiple levels and possible personalized paths for preventing and mitigating this symptom, which has such an impact on daily life.

Because fatigue profoundly impairs quality of life and is demonstrably mitigable, it emerges not as a secondary by-product of disease, but as a priority target for translational neuroscience.

## Cross-condition efficacy suggests a shared underlying mechanism

International literature reports that functional alterations within the sensorimotor system—especially involving the primary somatosensory (S1) and motor (M1) cortices—lead to inefficient intracortical communication and to a mismatch between perceived effort and actual motor output (Kuppuswamy, 2017). Such imbalance compromises the normal feedback between sensory input and motor control that sustains adaptive behavior (Tomasevic et al., 2013).

Fatigue is a transversal symptom, emerging across an extraordinary variety of conditions—from neurological and oncological diseases to post-infectious, metabolic, and psychosocial states. This ubiquity points to shared pathophysiological mechanisms (Kuppuswamy, 2022; Raizen et al., 2023): imbalance of regional excitatory and inhibitory neurotransmission, and consequent failure of behavioral adaptive ability. The existence of effective behavioral interventions—most stable being physical activity, mindfulness, yoga, and cognitive-behavioral therapy—confirms that this vulnerability can be mitigated through training the brain–body system toward restored coherence. In this context, where the same symptom arises independently of the underlying medical or non-medical condition and can be mitigated by the same interventions across contexts, fatigue is suggested to converge on a common neurophysiological substrate, rather than reflecting disease-specific damage.

Ultimately, fatigue deserves systematic attention as an early and transversal signal of imbalance, not a by-product of disease. Neuromodulation offers a concrete means to act upon this signal, bridging physiology, psychology, and environment. As research converges on shared mechanisms and personalized applications, neuromodulation can become a cornerstone of integrated multidisciplinary strategies—a precision tool to sustain recovery, by recovery a dysregulation of brain–body feedback loops, in which disrupted synchrony and altered excitation–inhibition balance impairs adaptive behavioral regulation.

## Neuromodulation as a physiological primer

Within this framework of pathways and tools, non-invasive neuromodulation stands out for its ability to make a fundamental contribution by directly intervening on the brain networks whose imbalance is known to be at the root of fatigue. In particular, transcranial direct current stimulation (tDCS) can selectively modulate the excitability of cortical circuits, facilitating the re-establishment of physiological synchrony sustaining recovery (Porcaro et al., 2019). In fact, neuromodulation offers a complementary interface with established behavioral therapies. Physical activity, mindfulness, yoga, and cognitive-behavioral therapy all rely on intact neural adaptability to be effective. When this adaptability is compromised—as in chronic neurological or post-infectious fatigue—neuromodulation can act as a physiological primer, restoring the cortical conditions necessary for successful learning and training. In this sense, it should not be viewed as an alternative but rather as an enabling support to behavioral and psychological interventions.

More than a decade of clinical evidence in MS paves the way to an effective, evidence-based treatment option (Tecchio et al., 2014; Cancelli et al., 2018; Gianni et al., 2021; Uygur-Kucukseymen et al., 2023). The following section of this Perspective is dedicated to reviewing the evidence in the condition of MS and outlining future directions for neuromodulation as emerging technology for Global Health and then also as a cornerstone in fatigue relief in diverse medical or non-medical conditions (World Health Organization - WHO, 2025).

Looking forward, the implications are broader. If fatigue represents a final common pathway of disrupted brain–body feedback, then restoring this loop through behavioral interventions and neuromodulation should benefit individuals independently of the condition that triggered the symptom.

Within this framework, it is useful to distinguish between mechanisms that primarily contribute to the emergence and maintenance of fatigue—such as disrupted network synchrony and excitatory–inhibitory imbalance—and mechanisms that represent actionable targets for neuromodulation, including neural adaptability, functional connectivity, and local neurodynamics, i.e., the ongoing time course of neuronal electrical activity.

The Faremus protocol (Fatigue Relief in Multiple Sclerosis) was conceived to rebalance this sensorimotor loop through bilateral stimulation of the somatosensory cortices (Tecchio et al., 2015; Porcaro et al., 2019). Each treatment cycle consists of five consecutive daily sessions, with 15 min of anodic tDCS per day delivered through personalized electrodes positioned over S1 with occipital cathode. The first demonstration of efficacy was reported by a randomized, sham-controlled design, where bilateral S1 tDCS significantly reduced self-reported fatigue in people with MS (PwMS) as measured by the mFIS (Tecchio et al., 2014). The improvement persisted beyond the stimulation period, suggesting that the intervention triggered enduring network-level changes rather than transient excitability shifts. The same personalized protocol was tested in PwMS, enriched by a automatized procedure for S1 electrode shaping (Cancelli et al., 2018). In both studies, S1 electrodes were tailored to each participant’s head morphology and cortical folding over both hemispheres. The clinical impact in the two PwSM groups confirmed that personalized, bilateral stimulation produced robust and sustained relief. Participants reported an average 26% reduction in fatigue. Faremus via home-based stimulation was demonstrated feasible, allowing patients to perform the five-day protocol under remote supervision with unchanged efficacy and safety (Tecchio et al., 2022). The most recent multicenter confirmation (Tecchio et al., 2025) involved coordinated recruitment across Italian institutions, including Rome and Genoa, consolidating Faremus as a reproducible, scalable intervention. The study reinforced the long-term persistence of benefits—often lasting several months—and contributed to the inclusion of tDCS for fatigue treatment.

**Figure 1:**
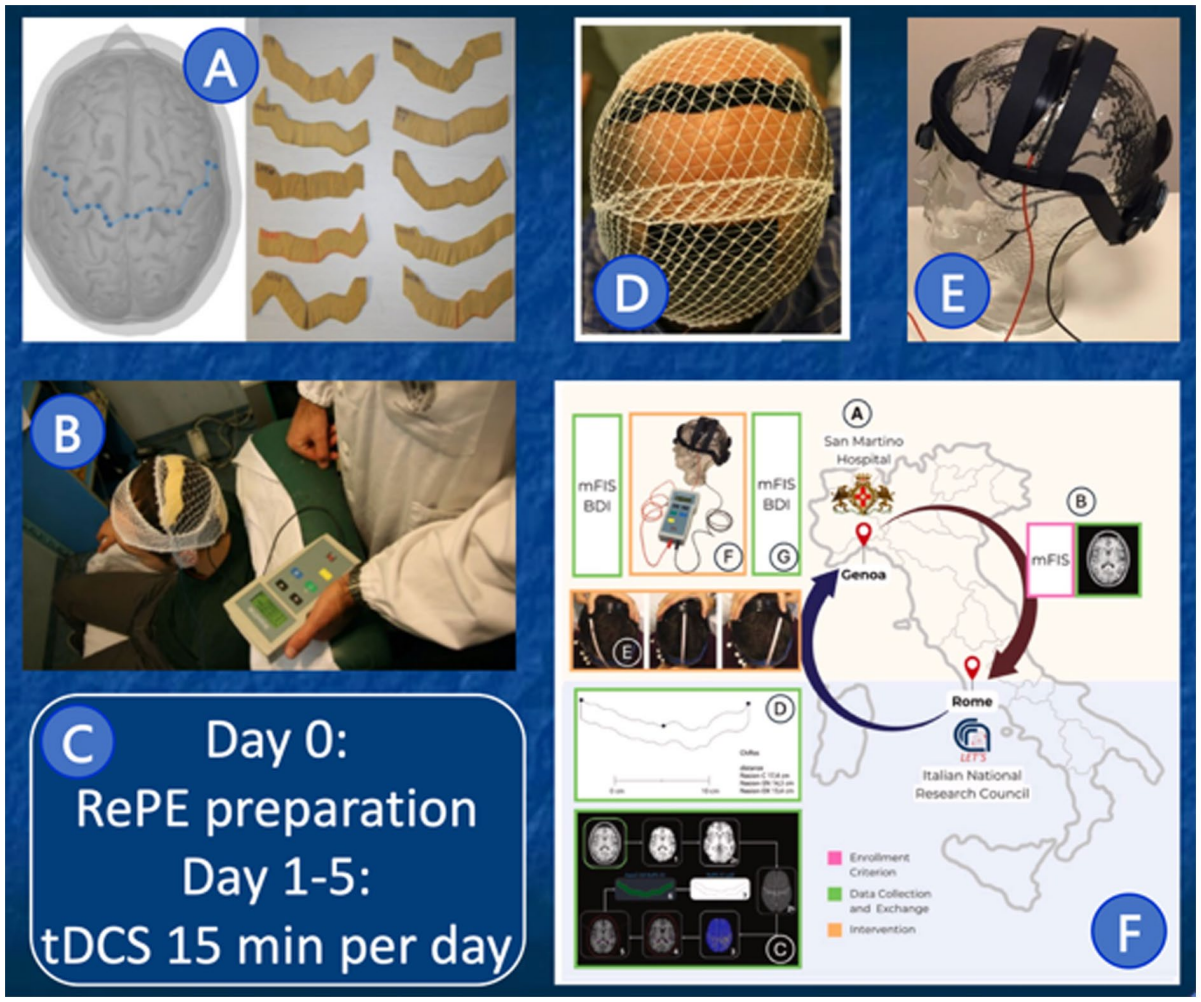
Faremus setup over time. As detailed in the dedicated text section, graphical representation of the fixed characteristics of Faremus **(C)** during the evolution of the methods and material for the S1 electrode **(A,B,D)** and the structure to ergonomically adapt the electrode to the head **(E)**, enabling home treatments and multicenter implementation **(F)**.

The development, which lasted more than 10 years, began with a very early phase in which electrodes were modeled directly on the patient’s head using a neuronavigation system and then fixed to the head with an elastic band (Figures 1A,B; Tecchio et al., 2014). What has remained constant from the initial investigation to today is the anodic target, consisting of the somatosensory representation region of the entire body bilaterally prepared once for each person, with an occipital (double-area) cathode, and a five-day treatment for 15 min per day (Figure 1C). A second step was to automate the electrode shape, which was made from a more user-friendly conductive silicone (Figure 1D; Cancelli et al., 2018). The qualitative leap occurred with the development of the electrode fastening system, eliminating the need for elastic bands, which were slightly annoying and necessarily used by a second person with a high level of training as a neurophysiological technician. We developed an adaptable helmet structure that allowed for precise repositioning of the electrodes between two successive sessions, even by the user and even in a home setting (Figure 1E; Tecchio et al., 2022). This same setup was used with a group from San Martino in Genoa, paving the way for the application of Faremus at a national level (Figure 1F; Tecchio et al., 2025).

Across these clinical investigations, converging results define the Faremus profile:

- Average improvement: about 26% decrease in mFIS scores relative to baseline.
- Duration of benefit: weeks to months after the five-day stimulation cycle.
- Safety: absence of serious adverse events; only mild, transient sensations under the electrodes in some person.
- Feasibility: minimal operator training; potential for home-based administration.

These features seem to advocate a decisive transition from exploratory research to clinical applicability.

Studies on the same subjects concurrent with Faremus clinical trials integrated neurophysiological recordings with clinical assessments before and after the intervention, supporting the role of neuronal network rebalancing in symptom mitigation. Improvements in sensorimotor functional connectivity and local ongoing time course of neuronal electrical activity—neurodynamics—accounted for about half of the observed fatigue reduction (Tecchio et al., 2015; Porcaro et al., 2019). These findings provided direct evidence that neuromodulation can effectively modify cortical dynamics underlying the symptom, confirming that fatigue improvement involves measurable restoration of physiological balances within sensorimotor circuits.

Beyond the Faremus experience, the international literature converges in identifying neuromodulation as one of the most promising interventions for MS-related fatigue. In their systematic review and meta-analysis, the Authors examined 44 randomized controlled trials applying non-invasive brain stimulation (NIBS) in MS (Uygur-Kucukseymen et al., 2023). Among the tested targets—left dorsolateral prefrontal cortex (DLPFC), right posterior parietal cortex (PPC), primary motor cortex (M1), and somatosensory cortex (S1)—the bilateral S1 stimulation used in Faremus emerged as the most reproducible and clinically effective, yielding consistent reductions in fatigue scores with a favorable safety profile. The Authors emphasized that, while other cortical targets such as the DLPFC showed variable and often short-lived results, protocols based on S1 modulation demonstrated higher effect size and persistence.

Earlier, other Authors had compared stimulation over the left DLPFC and right PPC, both involved in the fatigue-related network (Chalah et al., 2017). They found only partial improvement, limited by inter-study variability, and called for standardized, physiologically-driven approaches. This call was later addressed by the Faremus framework, whose design integrates neurophysiological rationale, reproducibility, and scalability.

More recently, a wide review was devoted to the exploratory and therapeutic potential of NIBS in MS fatigue (Ayache et al., 2022). Analyzing 27 studies, they confirmed that tDCS over bilateral S1 reliably reduces perceived fatigue, whereas repetitive transcranial magnetic stimulation (rTMS) or transcranial random noise stimulation (tRNS) protocols showed more heterogeneous outcomes. The review also underlined that effective protocols typically involve five consecutive daily sessions of 15–20 min at 1.5–2 mA, closely matching the Faremus design. Importantly, Ayache and Chalah highlighted the need to tailor electrode geometry and intensity to individual cortical anatomy, a principle realized in the Faremus personalized electrodes.

On the neurochemical level, an expanded review provided insights on neurotransmitter involvement in MS (Akyuz et al., 2023). Authors identified alterations in GABAergic and glutamatergic transmission as central to fatigue pathophysiology and suggested that neuromodulation may act by restoring this balance, enhancing adaptive plasticity and synaptic efficiency. This neurochemical evidence complements the network-level findings, reinforcing the interpretation of tDCS as a physiological tool rather than a merely symptomatic one.

In parallel, applying the GRADE (Grading of Recommendations Assessment, Development, and Evaluation) methodology, the strength of evidence supporting interventions targeting fatigue in MS was assessed (Gianni et al., 2021). Among all non-pharmacological strategies reviewed, Faremus ranked between *medium* and *high* in recommendability, confirming its clinical relevance and reproducibility across settings. The convergence of independent assessments—from single-site studies to international meta-analyses—establishes neuromodulation as a maturing, evidence-based therapy for MS fatigue.

Collectively, these data support a coherent interpretation: in subjects affected by MS fatigue mitigation arises from the capacity of neuromodulation to reinstate coherent neurodynamics in distributed networks. By facilitating synchrony between S1 and M1 and rebalancing inhibitory-excitatory coupling, the stimulation enhances the brain’s ability to integrate sensory feedback with motor planning.

The practical advantages of tDCS further strengthen its translational potential. It is painless, well tolerated, and devoid of serious side effects. Portable stimulators allow home-based use under remote supervision, a mode already validated within the Faremus trials (Tecchio et al., 2022). This accessibility expands clinical applicability and aligns with the broader movement toward self-managed, decentralized neurorehabilitation (Charvet et al., 2025).

The reproducibility of results, the durability of benefit, and the safety of home-based application confirm that tDCS can be feasibly integrated into routine clinical and rehabilitative settings. Its success in specific groups of people with multiple sclerosis—a paradigmatic condition in which fatigue precedes and remains independent of major disability—supports the view that early neuromodulatory intervention can help preserve participation and quality of life. This evidence paves the way for positioning neuromodulation as a cornerstone of fatigue relief across other medical and non-medical conditions, contributing to Global Health (World Health Organization - WHO, 2025). On these bases, neuromodulation starts moving beyond its exploratory phase. Through targeted modulation of the sensorimotor network, it offers an effective, safe, and reproducible method to alleviate fatigue. Its integration with behavioral therapies and other effective tools represents the next step toward precision fatigue relief, in which neuromodulation targets network-level neuronal balance, while neuronal adaptability and adaptive plasticity are supported by dedicated adjunctive interventions, acting in synergy to restore sustained well-being.

## Limitation and conclusion

Some limitations temper generalization of neuromodulation Faremus approach to mitigate fatigue. Heterogeneity of protocols—differences in electrode placement, current intensity, stimulation duration, and outcome measures—complicates direct comparison across studies. Sample sizes remain modest, and standardized biomarkers for predicting responders are lacking.

Future research should prioritize multicentre harmonization and incorporate objective indices of brain reactivity to stimulation, including neurophysiological and neuroimaging markers. In particular, most available neuromodulation studies, including those discussed here, primarily target subjective fatigue as it impacts daily functioning, rather than performance-based fatigability. While this focus reflects clinical relevance and feasibility, it limits the ability to disentangle central versus peripheral components of fatigue, as well as subjective versus objective dimensions. Although Faremus has been shown to induce behavioral changes (Tomasevic et al., 2013; Padalino et al., 2021) in addition to central neurodynamic effects (Porcaro et al., 2019), a deeper understanding of how neuromodulation-induced neural changes translate into behavioral outcomes will require the integration of conceptually grounded objective behavioral and central markers. The real-world translation of neuromodulation also requires integration into multidisciplinary care pathways and reimbursement frameworks, ensuring equitable access.

Despite these challenges, the convergence of evidence is striking: across independent studies and methodologies, non-invasive neuromodulation consistently mitigates fatigue. Among the available approaches, the Faremus protocol stands as the most validated model, linking clinical benefit to measurable neurophysiological change and demonstrating feasibility for large-scale, home-based application.

Awareness about the availability of methods to mitigate fatigue enhance the relevance of considering this symptom highly impacting everyday well-being. While our ultimate goal is contributing to build a network of multidisciplinary experts offering a suitable approach to overcome fatigue, here we present neuromodulation as a key tool when intra-cerebral processing does not allow proper behavioral adjustment.

## Data Availability

The original contributions presented in the study are included in the article/supplementary material, further inquiries can be directed to the corresponding author.
